# In vitro evaluation of fissure sealants’ wear under erosive, abrasive and erosive/abrasive conditions

**DOI:** 10.1007/s40368-022-00757-z

**Published:** 2022-09-28

**Authors:** B. Hamza, M. Sekularac, T. Attin, F. J. Wegehaupt

**Affiliations:** 1 Clinic of Orthodontics and Pediatric Dentistry, Center of Dental Medicine, University of Zurich, Plattenstrasse 11, 8032 Zurich, Switzerland; 2 Clinic of Conservative and Preventive Dentistry, Center of Dental Medicine, University of Zurich, 8032 Zurich, Switzerland

**Keywords:** Fissure sealants, Material wear, Fillers, Erosive challenge, Abrasive challenge

## Abstract

**Purpose:**

To evaluate and compare the wear of selected resin-based fissure sealants with different compositions properties under erosive, abrasive, and erosive/abrasive conditions.

**Methods:**

Forty-five samples of the following resin-based fissure sealants were prepared: Fissurit (fluoride free), Fissurit F (with fluoride), Fissurit FX (55 wt.% filler content), Grandio Seal (70 wt.% nano-filler content) and bovine enamel. Fifteen samples from each material were randomly allocated into three groups according to the wear condition they would be subjected to as follows: erosive condition (citric acid, 1 min, pH 2.3), abrasive condition (120 brushing strokes at 2 N, toothpaste slurry RDA value = 69), and erosive/abrasive condition (combination of both). The wear challenges were repeated six times each day for 10 days. The material wear was measured using a stylus profilometer. Kruskal–Wallis and Conover’s test was applied to compare the resulting material wear between the groups.

**Results:**

Under erosive conditions, Grandio Seal and Fissurit FX showed statistically significantly the least material wear. Under abrasive and erosive/abrasive conditions, Grandio Seal showed statistically significantly the least material wear. Fissurit F showed statistically significantly the highest material wear under abrasive and erosive/abrasive conditions, after dental enamel (*p* < 0.05).

**Conclusion:**

Higher filler content in sealants leads to better wear resistance. Incorporating fluoride into sealants seems to reduce their wear resistance at similar filler contents.

## Introduction

Pit and fissure sealants were introduced in the late 1960s (Ahovuo-Saloranta et al. 2017). Initially, sealants were applied on dental occlusal surfaces to prevent caries. However, their indication has further developed and are now used in treating initial caries lesions on both occlusal and approximal surfaces (Kashbour et al. 2020; Dorri et al. 2015). While clear indications for the application of sealants have been suggested by guidelines (Welbury et al. 2004; Wright et al. 2016), Splieth et al. (2010) reported that some university paediatric dentistry clinics apply sealants on routinely basis (during the first year of permanent molars’ eruption). Resin-based or glass ionomer sealants are the most available sealants in the market. Beside caries prevention and management, resin-based sealants have also been indicated in the prevention of erosive and abrasive tooth wear (Zhao et al. 2016; Wegehaupt et al. 2017).

Low wear resistance has always been one of the main failure sources (fracture, increased roughness) of resin-based—and glass ionomer—sealants. Mechanical challenges (toothbrushing, chewing) and acidic beverages were reported as the main source of sealants’ wear (Krüger et al. 2018; Faria et al. 2021). To overcome this shortcoming, resin-based sealants were loaded with fillers of different types, shapes and sizes (Faria et al. 2021). Another attempt to enhance the clinical performance of resin-based sealants was to load them with fluoride, which was found to reduce enamel demineralisation adjacent to the sealant (Kuşgöz et al. 2010).

As mentioned above, mechanical and acidic challenges were reported to be the main wear sources of resin-based sealants. This study was therefore carried out to evaluate and compare the wear of selected resin-based sealants with different compositions (Fissurit: no fluoride, Fissurit F: with fluoride, Fissurit FX: with fluoride and 55 wt.% filler content, Grandio Seal: fluoridated and 70 wt.% nano-filler content) under erosive, abrasive, and erosive/abrasive conditions. The null hypothesis was that there would be no differences between the tested sealants under each tested wear condition (abrasive, erosive, or erosive/abrasive).

## Materials and methods

Forty-five samples of enamel (positive control) and the 4 following sealant materials were prepared for this in vitro study: Fissurit (VOCO GmbH, Cuxhaven, Germany), Fissurit F (VOCO GmbH), Fissurit FX (VOCO GmbH), and Grandio Seal (VOCO GmbH). Table 1 shows the composition of each tested material.

**Table 1:** Tested material composition according to the manufacture

Material	Content	Properties
Fissurit	Bis-GMA (25–50%) UDMA (10–25%) 1,6-hexanediylbismethacrylate (10–25%) Fumed silica (5–10%)	Does not contain fluoride
Fissurit F	Bis-GMA (25–50%) UDMA (10–25%) 1,6-hexanediylbismethacrylate (10–25%) Fumed silica (5–10%) Sodium fluoride (2.5–5%)	Contains fluoride
Fissurit FX	TEGDMA (10–25%) UDMA (10–25%) Bis-EMA (5–10%) Bis-GMA (5–10%) Sodium fluoride (≤ 2.5%)	Contains fluoride 55 wt.% filler content
Grandio Seal	TEGDMA (10–25%) Fumed silica (5–10%) Bis-GMA (2.5–5%)	Contains fluoride 70 wt.% nano-filler content

*Bis-GMA* Bisphenol A-glycidyl methacrylate, *UDMA* Urethane dimethacrylate, *TEGDMA* Triethylene glycol dimethacrylate, *Bis-EMA* Bisphenol A diglycidyl methacrylate ethoxylated

The enamel samples were milled out of bovine incisor crowns using a diamond-coated trephine mill under constant water-cooling (Diameter = 3 mm). The sealant samples were prepared by applying the respective sealant material into a silicone mold (Diameter and depth = 3 mm). Two 1.5-mm layers of each sealant were applied and light cured (from the upper side) according to the manufacturer’s instructions (20 s at 1200 mW/cm^2^, Bluephase G2, Ivoclar Vivadent, Schaan, Liechtenstein). All samples were embedded in acrylic resin (Paladur; Heraeus Kulzer, Hanau, Germany) and then ground using 1200-, 2000- and 4000-grit carborundum papers inside a grinding machine (Tegramin-30, Stuers, Birmensdorf, Switzerland) under constant water-cooling. The 45 samples of the 5 tested materials (enamel and four sealants) were then randomised into 3 groups (*n* = 15) depending on the type of the wear condition they would be subjected to (Erosion, abrasion, erosion/abrasion). This created 5 groups of 15 samples in each of the three wear conditions types as shown in Table 2. The samples in all groups were stored for 3 weeks in tap water before they were subjected to the wear conditions. Baseline profilometric profile was recorded for each sample according to a standard procedure (Attin et al. 2009) using stylus profilometry (MFW-250, Perthometer S2; Mahr, Göttingen, Germany) and the groups were subjected to the planned wear condition. To serve as reference points for the profilometric recording, two parallel lines were scratched into the embedding material and parts of the tested material (enamel or sealant) were protected using an adhesive tape before subjecting the sample to the wear condition.

**Table 2:**
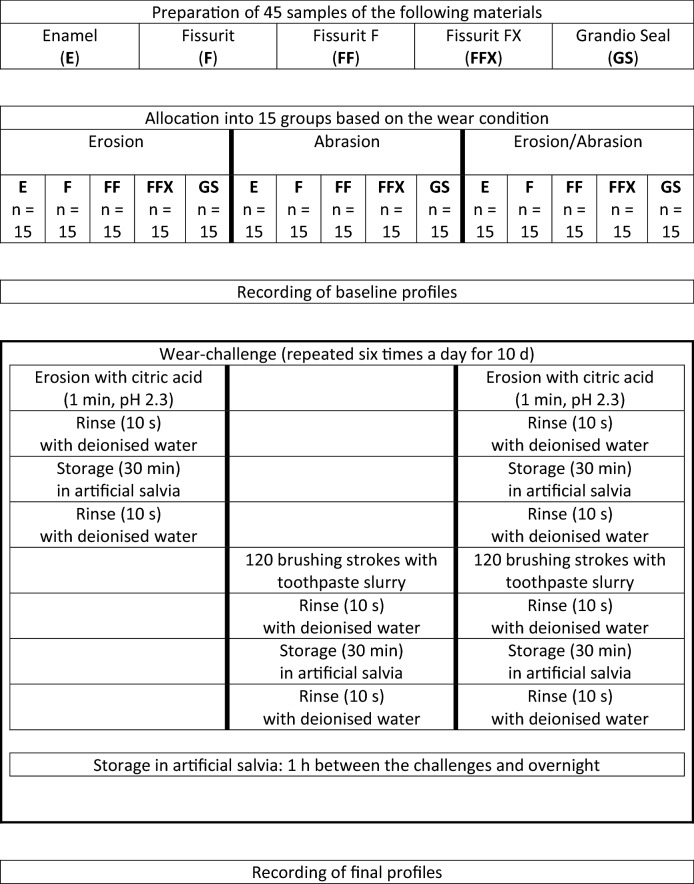
Study design

In the erosion groups, 5 ml of citric acid (pH 2.3, 25 ℃) was pipetted inside prefabricated containers (each container holding two samples) and kept without agitation for 1 min. Consequently, the samples were rinsed with deionised water (10 s), stored in artificial saliva (30 min, 37 ℃) (Klimek et al. 1982), and rinsed with deionised water again (10 s). This challenge was repeated six times a day (for 10 days) with 1-h intervals, during which the samples were stored in artificial saliva (37 ℃).

In the abrasion groups, the samples were subjected to 120 brushing strokes (at 2-N brushing force) inside a 6-place-brushing-machine using a slurry of Elmex Kariesschutz toothpaste (Colagte-Palmilive, Swidnica, Poland) and a medium-bristle toothbrush (Paro M43, Esro, Thalwil, Switzerland). Consequently, the samples were rinsed with deionised water (10 s), stored in artificial saliva (30 min, 37 ℃), and rinsed with deionised water again (10 s). This challenge was repeated six times a day (for 10 days) with 1-hour intervals, during which the samples were stored in artificial saliva (37 ℃).

In the erosion/abrasion groups, the aforementioned erosive challenge was firstly applied followed by the aforementioned abrasion challenge. Both challenges were repeated six times a day (for 10 days) with 1-h intervals, during which the samples were stored in artificial saliva (37 ℃). The samples were always stored in artificial saliva (37 ℃) overnight. After 10 days of running the wear challenges, final profiles were recorded and the material loss was determined as described before. The resulting material wear was calculated by superimposition of the baseline profile with the final profile. A prefabricated jig was used to ensure the minute positioning of the samples inside the profilometer for baseline and final profile recording. The abovementioned engraved scratches and the protected areas (underneath the adhesive tape) served as references. Five parallel profiles (distance between profiles = 250 µm, recording accuracy = 40 nm) were recorded for each sample. The mean of these five profiles was calculated and eventually used as the observed material wear of the respective sample. Table 2 summarises the study design.

### Statistical analysis

The mean and standard deviation (SD) of the erosive, abrasive, and erosive/abrasive wear for each material under each wear condition was calculated for each group. Kruskal–Wallis analysis was applied to detect any significant difference regarding the material wear under each condition. If a significant difference in the material wear was detected, Conover’s test was conducted for pairwise comparisons between the tested materials within the same wear condition. The significance level was set at 95% and the resulting *p* value was adjusted for multiple tests according to Holm. Data were analysed using the statistical program R (The R Foundation for Statistical Computing; Vienna, Austria.

## Results

The mean (± SD) of the calculated material wear of each tested material under the different wear conditions are presented in Table 3. Under erosive conditions, Grandio Seal and Fissurit FX showed the least wear compared to all other materials (*p* < 0.05). Under abrasive and erosive/abrasive conditions, Grandio Seal showed the least wear compared to all other groups (*p* < 0.0001). Enamel always showed the highest wear regardless of the applied wear condition.

**Table 3:** Wear of the investigated sealants and enamel under erosive, abrasive and erosive/abrasive conditions

	Erosion	Abrasion	Erosion/Abrasion
Fissurit (fluoride free)	− 0.08 ± 0.07 (A)^a^	0.60 ± 0.15 (A)	0.45 ± 0.10 (A)
Fissurit F (with fluoride)	− 0.09 ± 0.04 (A)	0.85 ± 0.19 (B)	0.62 ± 0.10 (B)
Fissurit FX (55 wt.% filler content)	− 0.04 ± 0.04 (B)	0.38 ± 0.10 (C)	0.36 ± 0.08 (C)
Grandio Seal Seal (70 wt.% nano-filler content)	− 0.02 ± 0.02 (B)	0.02 ± 0.03 (D)	0.05 ± 0.05 (D)
Enamel	9.77 ± 0.99 (D)	0.64 ± 0.30 (A)	11.62 ± 2.75 (E)

^a^Mean (± SD) of the material wear under each tested wear condition (in µm). Similar capital letters indicate no statistical significant difference between the tested materials within the same wear condition (read vertically)

## Discussion

In a recent review, Faria et al. (2021) reported that mechanical solicitations and the presence of acidic substances (e.g., citric acid, lactic acid and other acid beverages) were common conditions leading to degradation of fissure sealant materials. The present study was carried out to evaluate and compare the wear of selected sealant materials—with different properties—under various conditions (erosion, abrasion, and erosion/abrasion). The tested materials acted differently under each wear condition with Grandio Seal (contains fluoride and high content of nano-fillers) presenting the least wear under all wear conditions. The null hypothesis of the present study has, therefore, to be rejected.

The sealant samples were prepared and subjected to the wear conditions independently from enamel in this study (i.e., without applying them into actual pit and fissures or a smooth tooth surface). This was also the case in earlier studies, which pursued a same study question as the present one (Asefi et al. 2016; Krüger et al. 2018; Sangpanya et al. 2021). It could be argued that applying the sealants into pit and fissures would have better addressed the clinical situation. However, the present method aimed to standardise the samples, which would have been difficult if sealants had been applied into different teeth (different anatomy for each sample). The samples were brushed for 7200 strokes throughout the whole experiment using a slurry of commercially available toothpaste with an RDA value of 69 (Hamza et al. 2020) and a medium-bristle toothbrush. The amount of brushing strokes corresponds to slightly more than 1-year clinical brushing time when teeth are brushed twice daily for 2 min (Creeth et al. 2009). It could be assumed that different material wear might have resulted if a different toothpaste (e.g., with different RDA value) or a different toothbrush (e.g., different bristle stiffness) had been used (Hamza et al. 2021). Regarding the erosive challenge, the samples were eroded with citric acid at 25 ℃ for 1 min/cycle in this study. This duration is within the recommendation set by Wiegand and Attin (2011) for the extra oral acidic challenges not to exceed 2 min for each cycle due to the absence of saliva. It could, however, be argued that acidic beverages are not usually consumed at such temperature. Less erosive material wear would have been observed if the acidic challenge had been carried out at a lower temperature (Steiger-Ronay et al. 2018). On the other hand, the selected challenge conditions helped evaluating and comparing the performance of the tested materials under rather hard erosive and abrasive conditions.

Fissurit and Fissurit F showed similar material wear under erosive conditions. However, Fissurit F showed statistically significantly more material wear than Fissurit under abrasive and erosive/abrasive conditions. As adding sodium fluoride seems to be the only difference between those sealants (Table 1), a question might be raised whether the fluoride addition is responsible for the observed reduction in wear resistance for Fissurit F. Two methods of incorporating fluoride into sealants have been reported in the literature: Either by adding fluoride to non-polymerised resin in the form of soluble fluoride salt, or by chemically binding an organic fluoride compound to the resin. In the first method, the fluoride salt dissolves after applying the sealant, which is also connected to an in situ weakening of the sealant’s surface (Sangpanya et al. 2021; Morphis et al. 2000). As for the present tested materials, it is not declared which method was used to incorporate fluoride into Fissurit F. Nevertheless, it could be speculated that the aforementioned fluoride incorporation method might have played a role in the reduced wear resistance of Fissurit F observed in this study. As a matter of fact, this speculation (dissolve of fluoride salts) might also explain the negative values observed for the sealants under erosive conditions. In other words, it could be assumed that dissolved fluoride salts might have precipitated on the surface of the samples forming a film or an irregularity interpreted as negative values (gain instead of wear) in the profilometric analysis. Again, this is only a speculation, especially that such negative values were also observed for the fluoride-free tested sealant “Fissurit”. Regardless, this in vitro reduction in the wear resistance should in no way be interpreted as clinical superiority of fluoride-free sealants over fluoride-containing ones.

Both highly filled tested sealants (Fissurit FX and Grandio Seal) showed similar wear under erosive conditions. Under abrasive and erosive/abrasive conditions, however, Grandio Seal showed statistically significantly less wear than Fissurit FX—and all other tested materials. This observed difference is most probably attributed to the higher filler content inside Grandio Seal (leading to increased material hardness) and corroborates the findings of Souza et al. (2016), which indicated a decisive effect of the volume content of fillers on the mechanical and tribological properties of resin-based materials. In contrast to the present study, Sangpanya et al. (2021) found that unfilled sealants underwent less material wear under abrasive conditions than filled sealants (filler content range was 40 to 60 wt.%). The fact that the latter study utilised a different abrasive challenge than the present study (different toothpaste slurry (RDA = 40), 48,000 brushing strokes, 3-N brushing force, different protocol for profile recording, etc.) does not actually help explaining the contrast findings. Pardia et al. (2008) observed no differences in the material wear between unfilled and filled sealants under abrasive conditions. The authors—also not having expected this result—attributed their finding to the same polymeric matrix presented in the tested filled and unfilled sealants. They speculated that the matrix might play a more important role than the filler content in wear resistance. The chemical composition of the polymeric matrix was indeed mentioned as a parameter affecting the wear resistance of resin-based sealants in the recent review of Faria et al. (2021), without referring to its relative importance compared to the filler content. The last review also concluded that unfilled sealants show higher surface damage than filled sealants, corroborating the findings of the present study.

The fact that the control group (enamel) showed significantly higher erosive and erosive/abrasive wear than all tested sealants was rather expected and corresponds with the well-known clinical manifestation of eroded teeth that are filled with resin-based restorations (i.e., the observed elevated and disintegrated restoration margins) (Attin and Wegehaupt 2014). Erosive attacks soften the enamel and make it highly susceptible to physical forces, which also explains the higher enamel wear under erosive/abrasive condition in comparison to only erosion in the present study (Shellis and Addy 2014). Although much less than enamel, resin-based materials could also be attacked and degraded by acids. Yu et al. (2009) reported that this resin degradation is attributed to damages in the polymer matrix and loss of fillers. Similar to the present study, the latter study also reported higher enamel wear under erosive and erosive/abrasive conditions than the tested resin-based materials (Yu et al. 2009).

One of the limitations of the present study is the fact that no clinical relevance could be drawn from it. For instance, it cannot be predicted if the observed higher material wear in fluoride-releasing sealants compared to fluoride-free sealants would have any effect on the clinical performance of both materials. Another limitation of the here-used laboratory procedure includes the lack of thermal challenging of the tested materials (i.e., altering the temperature between 5 and 55 ℃ as an imitation of consuming cold and hot beverages). A further limitation can be seen in the high number of abrasive challenges performed per day and the ratio of erosive to abrasive challenges. Under clinical conditions, toothbrushing is normally performed about two times per day. Therefore, the used number of abrasive conditions results in an overestimation of the resulting abrasive and erosive/abrasive wear. However, as the here gained data are only compared within the present study, such an overestimation seems to be acceptable.

## Conclusions

Based on this study and within its limits, it could be concluded that different sealant materials with different properties exhibit different wear behaviour under the tested in vitro wear conditions. Higher contents of fillers could add to the wear resistance of fissure sealants under erosive, abrasive, and erosive abrasive conditions, while fluoride-release could reduce the wear resistance under abrasive and erosive/abrasive conditions.

## Data Availability

Data available on request from the corresponding author.

## Code availability

Non-applicable.
